# Mediterranean Diet vs. Time-Restricted Eating: Effects on Circadian Rhythm and Appetite Markers in Female Patients with Multiple Sclerosis—A Pilot Study

**DOI:** 10.3390/healthcare14152266

**Published:** 2026-07-24

**Authors:** Elif Gökçe İnbaşı, Nihan Çakır Biçer, Dilek İşcan, Ergül Bayram, Perim Fatma Türker

**Affiliations:** 1 Department of Nutrition and Dietetics, Bor Faculty of Health Sciences, Nigde Omer Halisdemir University, 51700 Nigde, Türkiye; einbasi@ohu.edu.tr; 2 Department of Nutrition and Dietetics, Graduate School of Health Sciences, Acibadem Mehmet Ali Aydınlar University, 34752 Istanbul, Türkiye; 3 Department of Molecular Gastroenterology and Hepatology, Institute of Gastroenterology and Hepatology, Kocaeli University, 41001 Kocaeli, Türkiye; nihancakir@gmail.com; 4 Department of Neurology, Baskent University Adana Hospital, 01120 Adana, Türkiye; dilekiscann@gmail.com; 5 Department of Medical Biochemistry, Nigde Omer Halisdemir Research and Training Hospital, 51200 Nigde, Türkiye; eyaylagul@hotmail.com; 6 Department of Nutrition and Dietetics, Faculty of Health Sciences, Acibadem Mehmet Ali Aydınlar University, 34752 Istanbul, Türkiye

**Keywords:** multiple sclerosis, Mediterranean diet, time-restricted eating, circadian rhythm, sleep, appetite

## Abstract

**Background/Objectives**: Multiple sclerosis is frequently associated with sleep disturbances and circadian rhythm disruptions. This study evaluated the effects of the Mediterranean diet (MedDiet) (*n* = 17) and time-restricted eating (TRE) (*n* = 17) on sleep, chronotype, and appetite-related parameters in females with relapsing–remitting MS. **Methods**: In this 12-week randomized comparative pilot trial, 34 female patients with RRMS were randomly assigned to either a MedDiet or TRE (16:8 protocol) group. Individualized eucaloric diet plans were provided to maintain baseline weight. Biochemical parameters (fasting glucose, lipid profile, leptin, and ghrelin levels), anthropometric measurements, the Pittsburgh Sleep Quality Index (PSQI), the Morningness and Eveningness Sleep Questionnaire (MEQ), and dietary intake were assessed at baseline and week 12. **Results**: Both groups showed significant reductions in body mass index, waist and hip circumference, although no statistically significant changes were observed in reported energy intake. In the MedDiet group, LDL (101.88 vs. 99.12 mg/dL) and total cholesterol (178 vs. 173 mg/dL) decreased before correction for multiple comparisons (*p* < 0.01); however, these findings did not remain statistically significant after multiple-comparison correction. Leptin and ghrelin showed no significant changes in either group. Both groups increased dietary protein, fiber, and water intake and decreased fat intake, while carbohydrate intake remained unchanged. MEQ scores increased only in the MedDiet group (*p* = 0.035). Mixed-design ANOVA revealed a significant time effect on PSQI scores independent of diet type (F = 11.370, *p* = 0.002), although this effect could not be attributed to a specific dietary intervention. **Conclusions**: Both diets were associated with changes in anthropometric measures. The MedDiet was associated with increased MEQ scores and trends toward favorable changes in lipid parameters, suggesting a shift toward a more morning-oriented chronotype rather than a direct effect on circadian rhythm. No significant changes were observed in sleep quality or appetite-related hormones. Further studies with larger samples and longer durations are needed.

## 1. Introduction

Multiple sclerosis (MS), an autoimmune disease of the central nervous system (CNS), is characterized by chronic inflammation, demyelination, and neuronal loss of unknown etiology [1]. With an estimated global prevalence of 2.5 million, MS predominantly affects young adults, particularly females [2,3]. The relapsing–remitting MS (RRMS) accounts for approximately 85% of all cases [4]. The exact etiology of MS remains unclear despite extensive research. However, it is widely accepted that a complex interaction between genetic predisposition and environmental factors contributes to its development. Environmental factors, including some infections, low vitamin D levels, smoking, nutrition, and obesity, have been associated with an increased risk of MS, with diet emerging as a potential modifiable risk factor [5].

Dietary patterns are known to link gut microbial balance and neuroinflammation; however, the current findings are inconsistent regarding the role of specific dietary components in MS progression [6,7,8,9,10]. Despite these inconsistencies, the Mediterranean diet (MedDiet), which is rich in anti-inflammatory and antioxidant compounds, has been associated with reduced systemic inflammation and improved neurological outcomes [10,11,12,13]. However, studies specifically investigating the effects of MedDiet on MS remain lacking.

Along with MedDiet, energy restriction has also been reported to positively affect brain health; however, both are challenging to maintain in the long term [14,15,16]. MedDiet and energy restriction have been associated with a reduced risk/prevalence of metabolic and neurological diseases [17]. The following are the three types of intermittent fasting (IF) diets: time-restricted eating (TRE), alternate-day fasting (ADF), and periodic fasting (PF). ADF involves alternating between fasting and non-fasting days, whereas PF encompasses abstaining from or restricting food intake for 2 days/week with unrestricted eating on the remaining 5 days. TRE limits food intake to an 8 h window each day, followed by16 h6-h fasting period. Fasting periods have been shown to potentially improve neurological and cognitive functions through metabolic, cellular, and circadian mechanisms and may help prevent brain-related disorders [18]. Through circadian clock stabilization and immunological and metabolic rhythm restoration, chronotherapeutic approaches, including TRE, are believed to help individuals with MS and possibly stop disease progression [19]. There is increasing evidence that MS is associated with the disruption of circadian rhythms, which represent physical, mental, and behavioral patterns that oscillate repeatedly every 24 h [20]. However, whether circadian disruption and MS-related fatigue are caused by disease-associated inflammation and neurodegeneration or are a contributing factor to disease onset remains unclear [19,20,21].

In addition to circadian and metabolic pathways, appetite-related hormones such as leptin and ghrelin, which are influenced by diet and fasting, may play roles in neuroinflammation. Recent studies have indicated that in addition to being produced by adipocytes, leptin receptors are expressed in various regions of the CNS. Research suggests that leptin contributes to the regulation of neuronal survival and neural development [22,23,24]. Moreover, leptin has demonstrated neuroprotective properties in animal models of neurological disorders and demyelination [25]. Ghrelin, an orexigenic peptide hormone, is derived from the intestine and mainly regulates growth hormone secretion, food intake, and energy homeostasis. Ghrelin has been implicated in several higher-order brain functions, including memory, reward, mood, and sleep [26]. Considering the potential role of diet in modulating neuroinflammation, circadian regulation, and appetite hormones in MS, this study aimed to evaluate the effects of TRE and the MedDiet on sleep quality, circadian rhythm, and appetite-related parameters, particularly serum leptin and ghrelin levels, in females aged 18–65 years with a diagnosis of RRMS. It was hypothesized that the MedDiet and TRE would differ in their effects on sleep quality, circadian rhythm characteristics, and appetite-related hormonal parameters, particularly serum leptin and ghrelin concentrations, in women with RRMS.

## 2. Materials and Methods

This study was conducted as a two-arm randomized, parallel-group comparative pilot trial between August 2024 and March 2025 at the Neurology Outpatient Clinic of Nigde Omer Halisdemir Training and Research Hospital. The study was approved by the Acibadem Mehmet Ali Aydinlar University Medical Research Ethics Committee (approval number: ATADEK-2024-7/286; Date: 2 May 2024) and was conducted in accordance with the Declaration of Helsinki. Written informed consent was obtained after the aims and action plan of the study were thoroughly explained to each participant. This study was prospectively registered at ClinicalTrials.gov (Identifier: NCT06546033). The study design, conduct, and reporting were completed and presented in accordance with the CONSORT guidelines.

### 2.1. Study Design

The following were the inclusion criteria for this study: diagnosed with RRMS for at least 2 years, aged 18–65 years, being female, not being in a relapse period and having no relapses in the past 6 months, not being in the menopausal period, and having an Expanded Disability Status Scale (EDSS) score of <3.5, which is a broadly used and internationally validated tool for evaluating disability and impairment in patients with MS [27].

The following were the exclusion criteria: a diagnosis of any autoimmune disease other than MS, having any neurological disease other than MS, being in a relapse period, having other forms of MS, being newly diagnosed, having an EDSS score of ≥3.5, having a physical or mental disability, having a history of cancer, using vitamin or mineral supplements, not regularly using medications prescribed by a physician for MS treatment, having adhered to the MedDiet or TRE in the last 3 months, being pregnant or lactating, being in the menstrual cycle at the beginning or end of the study, having communication difficulties, having a body mass index (BMI) outside the range of 18.5–29.9 kg/m^2^, having lost ≥5% body weight in the last month, or aged < 18 or > 65 years.

A power analysis was conducted using the G*Power software (Version 3.1.9.7; Heinrich Heine University Düsseldorf, Düsseldorf, Germany) to determine the sample size; calculations were performed at a 95% confidence level (*p* < 0.05). Since the study aimed to examine the time × diet type interaction between two parallel groups, the power analysis was conducted within the framework of an ANOVA with repeated measures (within-between interaction). The analysis was conducted under the following assumptions: two groups, two measurement time points, a moderate effect size (f = 0.25), a correlation between repeated measures of r = 0.50, and α = 0.05. The analysis indicated that a total of 34 participants (17 per group) would provide approximately 80.7% statistical power; this percentage is considered sufficient [28].

**Figure 1:**
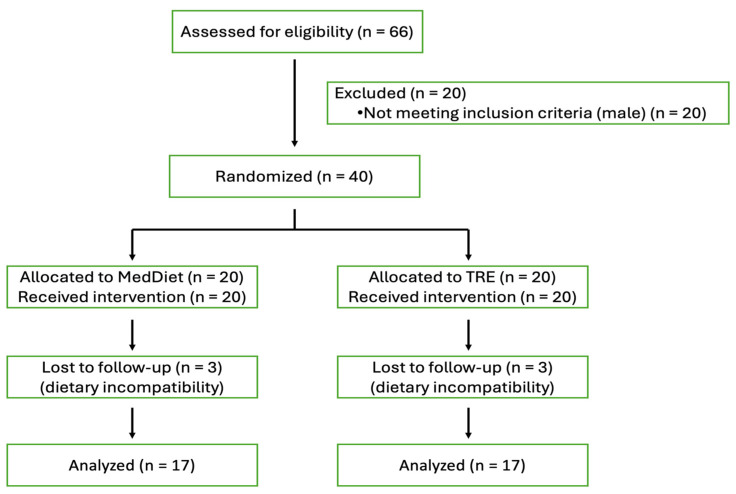
CONSORT flow diagram of patient enrollment, allocation, follow-up, and analysis.

This study was conducted as a two-arm randomized, parallel-group comparative pilot trial. All participants met the 2017 McDonald criteria for RRMS diagnosis prior to enrollment [29]. A total of 66 individuals were screened for eligibility. As the study was designed to include only female participants in order to minimize sex-related variability in appetite regulation, hormonal responses, and circadian parameters, 20 male patients were not included at the screening stage. Following screening and allowing for an anticipated dropout rate of approximately 20%, 40 eligible female patients were enrolled. Participants were randomly assigned to either the Mediterranean diet or time-restricted eating intervention group. During the intervention, six participants (three from the MedDiet group and three from the TRE group) withdrew due to dietary incompatibility. Ultimately, 34 participants completed the study and were included in the final analysis. The patient selection process is illustrated in Figure 1.

Participants were randomly assigned to either the MedDiet or TRE group using a computerized random number generator after enrollment had been completed. All eligible participants were identified and enrolled before randomization, and the random allocation sequence was generated by a member of the research team who was not involved in delivering the dietary interventions. Therefore, group assignment did not influence participant recruitment or eligibility assessment. Due to the nature of the dietary interventions, participants and dietitians could not be blinded to group allocation. Biochemical analyses were performed by an independent laboratory (SANCAR-LAB) that was blinded to group assignments. Statistical analyses were conducted by a researcher who was aware of the group assignments.

Each group followed their assigned dietary intervention for 12 weeks. Both groups were provided with individualized diet plans that were designed to be eucaloric, with estimated energy requirements calculated using the Harris–Benedict equation and the intention of maintaining baseline body weight. The macronutrient composition of the diets consisted of 55–60% carbohydrates, 15–20% protein, and 25–35% fats. Dietary compliance was monitored through 24 h dietary recalls collected at baseline and once weekly on a randomly selected day throughout the 12-week intervention (13 times in total).

**Figure 2:**
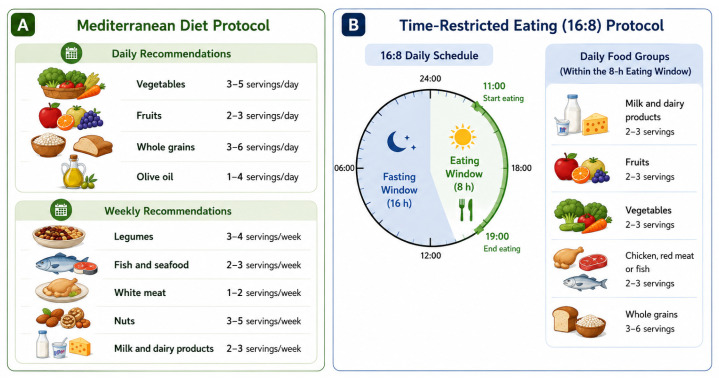
Schematic representation of the dietary interventions applied during the study. (**A**) Mediterranean diet protocol emphasizing plant-based foods, olive oil, and weekly consumption of selected food groups. (**B**) Time-restricted eating protocol consisting of an 8 h eating window and 16 h fasting period.

In the MedDiet group, the dietary model included daily servings of 3–5 servings of vegetables, 2–3 servings of fruits, 3–6 servings of whole grains, and 1–4 servings of olive oil; weekly servings of food groups included 3–4 servings of legumes, 2–3 servings of fish and seafood, 1–2 servings of white meat, 3–5 servings of nuts, and 2–3 servings of milk and dairy products [30]. In the TRE group, participants were instructed to consume all meals within an 8 h window each day (16:8 protocol), starting no later than 11:00 a.m. The daily TRE dietary model included 2–3 servings of milk and dairy products, 2–3 servings of fruits, 2–3 servings of vegetables, 2–3 servings of chicken, red meat, or fish, and 3–6 servings of whole grains. During the 16 h fasting period, participants were allowed to consume only water, unsweetened tea, or black coffee [31]. A schematic overview of the Mediterranean diet and time-restricted eating protocols used in the study is presented in Figure 2.

### 2.2. Data Collection Tools

Data were collected using several tools, including a demographic questionnaire, anthropometric measurements, the Pittsburgh Sleep Quality Index (PSQI), the Morningness and Eveningness Questionnaire (MEQ), 24 h food consumption records, and biochemical analyses. The demographic data included age, marital and education status, occupation, lifestyle habits, and age at diagnosis. The PSQI and MEQ were administered at baseline and after the 12-week dietary intervention to assess changes in circadian rhythm and sleep quality. Dietary food intake was evaluated using 24 h food consumption records collected by a specialized dietitian. To support dietary compliance, participants were followed up with weekly phone calls throughout the 12-week intervention, during which additional dietary records were obtained.

#### 2.2.1. Anthropometric Measurements

Anthropometric measurements were conducted by a specialist dietitian at two different times: before (pre-diet) and after (post-diet) the dietary intervention. Body weight (kg) was measured using bioelectrical impedance analysis (BIA) (Tanita BC-601). Height (cm) was measured using a portable stadiometer. BMI was calculated as weight (kg) divided by height squared (m^2^) and classified according to the World Health Organization [32]. Waist circumference (WC) and hip circumference (HC) were measured using a nonflexible tape measure with 1 mm precision. HC was measured using the iliac crest and the widest diameter of the hips. WC was measured between the costal margin and the iliac crest, the narrowest part of the trunk.

#### 2.2.2. Pittsburgh Sleep Quality Index (PSQI)

The PSQI was developed by Buysse et al. in 1989 to assess sleep quality and sleep disturbance severity over the past month [33]. The PSQI consists of 19 items and 7 subscales. The PSQI assesses seven subdimensions—subjective sleep quality, sleep latency, sleep duration, habitual sleep efficiency, sleep disturbance, sleep medication use, and daytime dysfunction—based on the respondent’s answers to 19 questions. Each item is scored on a scale ranging from 0 (no distress) to 3 (severe distress). The sum of the subscale scores provides the total PSQI score, varying from 0 to 21 points. A total score of 5 or less indicates “good” sleep quality. The Turkish adaptation of the PSQI was performed by Agargun et al. in 1996 [34]. Cronbach’s alpha value of the scale was 0.80 [34]. In this study, the PSQI had Cronbach’s alpha coefficients of 0.803 and 0.779 at pre- and post-diet, respectively.

#### 2.2.3. Morningness and Eveningness Questionnaire (MEQ)

The MEQ was developed by Horne and Ostberg in 1975 to assess individual chronotype preferences [35]. The MEQ is a 19-item, one-dimensional 5-point Likert-type scale. Based on the score, the participants were classified into different chronotype categories. The total score ranges from 0 to 86. Based on the total scores obtained for the 19 items, a score range of 70–86 indicates a “definitely morning type,” 59–69 indicates a “moderately morning type,” 42–58 points is classified as “neither type,” 31–41 points as “moderately evening type,” and 16–30 points as “definitely evening type,” resulting in five distinct circadian type classifications. The Turkish adaptation of the MEQ was performed by Punduk et al. in 2005 [36]. For Turkish validity, the questionnaire was administered a second time to the same people under the same conditions, 15–20 days later, in accordance with a time interval long enough to prevent significant recall, but short enough not to allow significant changes in the trait to be measured. The same individual evaluated the questionnaire results. Cronbach’s alpha values of the scale were 0.785 and 0.812 in the first adaptation study and second application study, respectively [36]. In this study, Cronbach’s alpha coefficients of the MEQ were 0.828 and 0.851 at pre- and post-diet, respectively.

#### 2.2.4. Twenty-Four-Hour Dietary Recall

To assess the daily energy and macronutrient intakes of the participants, food consumption records were obtained by a specialist dietitian using the 24 h dietary recall method. Dietary follow-up was conducted for 12 weeks, and a 24 h food consumption record was collected on a random day of each week following diet initiation. Individuals were informed about the portion sizes and food groups, and training on food consumption recording was provided to instantly record daily food consumption. To determine the average intake for both the baseline and 12-week periods, daily energy and macro- and micro-nutrient intake values were analyzed using a computer-assisted nutrition and information system program (BeBiS v.9).

#### 2.2.5. Biochemical Analyses

Blood samples were analyzed for high-density lipoprotein (HDL) cholesterol, low-density lipoprotein (LDL) cholesterol, total cholesterol, triglyceride (TG), fasting serum glucose (FSG), leptin, and ghrelin levels. The coefficients of variation (intra-assay CV) for FSG in the study were 0.7% for the low range and 0.8% for the high range; the inter-assay CV was 1.1% for the low range and 1.3% for the high range. The common reference range is 74–106 mg/dL. FSG levels in serum samples were measured using the “hexokinase” spectrophotometric method. The coefficients of variation (intra-assay CV) for serum HDL are 0.4% for low levels and 0.6% for high levels; the inter-assay CV is 1.4% for low levels and 1.6% for high levels. The common reference range is 35–55 mg/dL. HDL levels in serum samples were measured using the “homogeneous enzymatic colorimetric test” spectrophotometric method. The coefficients of variation (intra-assay CV) for LDL are 0.5% for low levels and 0.6% for high levels; the inter-assay CV is 1.0% for low levels and 1.1% for high levels. The common reference range is 0–130 mg/dL. LDL levels in serum samples were calculated using the Friedewald formula. The coefficients of variation (intra-assay CV) for TG are 0.6% for low levels and 0.9% for high levels; the inter-assay CV is 1.6% for low levels and 2.0% for high levels. The common reference range is <150 mg/dL. TG levels in serum samples were measured using the “enzymatic colorimetric test” spectrophotometric method. The coefficients of variation (intra-assay CV) for total cholesterol are 0.6% for low levels and 0.8% for high levels; the inter-assay CV is 1.4% for low levels and 1.6% for high levels. The common reference range is 0–200 mg/dL. Total cholesterol levels in serum samples were measured using the “enzymatic colorimetric test” spectrophotometric method. The coefficient of variation (intra-assay CV) for leptin is <10%, and the inter-assay CV is <12%. Leptin levels were measured in serum samples using the “sandwich” ELISA method. The coefficient of variation (intra-assay CV) for ghrelin is <10%, and the inter-assay CV is <12%. Ghrelin levels were measured in serum samples using the “enzyme immunoassay technique” ELISA method. Leptin and ghrelin values were not reported in the insert; accordingly, these hormonal parameters were analyzed only within each group by assessing pre- to post-intervention changes.

Samples were collected at baseline and at the end of the 12-week intervention after at least 12 h of overnight fasting. Subsequently, the blood samples were centrifuged at 5000× *g* rpm for 5 min, and the isolated sera were placed in microtubes. The collected samples were stored at −80 °C for further analysis at the end of the study. Serum leptin and ghrelin levels were measured using commercial human ELISA kits (Algen Diagnostic Medical Ltd., Ankara, Türkiye; catalog numbers SEA084Hu and CEA991Hu) according to the manufacturer’s instructions. Leptin levels were quantified using a Human Leptin ELISA kit (Cloud-Clone Corp., Wuhan, China; catalog number SEA084Hu), which provides an analytical range of 0.156–10 ng/mL and a manufacturer-reported limit of detection (LOD) below 0.058 ng/mL. Ghrelin concentrations were measured using a Human Ghrelin ELISA kit (Cloud-Clone Corp., Wuhan, China; catalog number CEA991Hu), offering a quantification range of 123.5–10,000 pg/mL and LOD below 44.5 pg/mL. All laboratory measurements were performed in the same laboratory (SANCAR-LAB, Nigde, Türkiye).

### 2.3. Statistical Analyses

All statistical analyses were performed using IBM SPSS Statistics (version 27; IBM Corp., Armonk, NY, USA, 2020) and Jamovi (version 2.6.19.0; The jamovi Project, Sydney, Australia). The Shapiro–Wilk test was used to assess the normality of the data distributions for variables with sample sizes < 50. Normally distributed variables were presented as mean ± standard deviation (mean ± SD), whereas non-normally distributed variables were presented as median (interquartile range). Categorical variables were presented as frequency (*n*) and percentage (%). For between-group comparisons of demographic and clinical data, anthropometric measurements, biochemical markers, energy and macronutrient intake, and scale scores, the Mann–Whitney U test was applied for non-normally distributed data, whereas the independent samples *t*-test was used for normally distributed data. For within-group (pre- and post-intervention) comparisons of the same variables, the Wilcoxon signed-rank test was used for non-normally distributed data, whereas the paired samples *t*-test was employed for normally distributed data. For appetite-related hormones (leptin and ghrelin), change scores (Δ = post-intervention − baseline) were calculated and compared between groups using the independent samples *t*-test. Effect sizes were calculated as Cohen’s d for parametric tests and rank–biserial correlation (rb) for non-parametric tests. Ninety-five percent confidence intervals (95% CIs) were reported for all comparisons. Fisher’s exact test was used to compare categorical demographic characteristics and chronotype classifications between the diet groups when expected frequencies were <5. The Stuart–Maxwell homogeneity test was used to assess changes in chronotype classifications within groups. A mixed design ANOVA was conducted to examine the effects of time and diet type, as well as their interaction on study outcomes. The reliability of the PSQI and MEQ was evaluated using Cronbach’s alpha coefficients. The Benjamini–Hochberg (BH) approach, which is a more balanced method than the Bonferroni method for controlling the Type I error rate in multiple comparisons, was chosen. In this approach, rather than applying a fixed threshold to all tests, the tests are ranked by their *p*-values, and a different critical threshold is assigned to each one. This correction was applied only to tests that yielded *p* < 0.05 in the original analysis; since tests with *p* > 0.05 are already insignificant, the correction will not alter these results. A *p*-value of < 0.05 was considered statistically significant.

## 3. Results

Both the MedDiet and TRE groups consisted of 17 female participants diagnosed with RRMS. As shown in Table 1, there were no significant differences between the groups in socio-demographic characteristics, including age, age at diagnosis, marital status, education level, employment status, physical activity, smoking, and alcohol consumption at baseline (all *p* > 0.05, small effect sizes). These results indicate that the two groups were comparable in their baseline characteristics, suggesting that any differences observed in subsequent analyses could be attributed to the dietary interventions. The mean age of the participants in the MedDiet and TRE groups was 34.06 ± 8.55 and 34.65 ± 8.23 years, respectively (*p* > 0.05). The mean age at diagnosis in the MedDiet and TRE groups was 27 and 22 years, respectively. Almost all of the participants (*n* = 32, 94.1%) in both groups reported not consuming alcohol, and the proportion of smoking was also low (*p* > 0.05). In both groups, 64.7% (*n* = 11) of the participants performed regular physical activity (*p* > 0.05) (Table 1).

The comparison of pre- and post-diet anthropometric measurements according to the diet groups is presented in Table 2. Both the MedDiet and TRE groups showed significant reductions in body weight, BMI, WC, and HC from baseline to post-diet intervention (all *p* < 0.05).

The comparison of pre-diet and 12-week mean dietary energy and macronutrient intake according to the diet groups is shown in Table 3. In both groups, total reported energy intake did not change significantly (*p* > 0.05). Carbohydrate intake (g/day) also showed no significant change in either group, whereas protein intake increased, and fat intake decreased significantly (*p* < 0.05). In addition, water and fiber intake increased significantly in both groups (*p* < 0.05).

The comparison of pre- and post-diet biochemical markers according to the diet groups is presented in Table 4. In the MedDiet group, although a significant decrease in serum LDL and total cholesterol levels was observed following the 12-week dietary intervention (*p* < 0.05), these findings did not meet the threshold for statistical significance after adjustment for multiple comparisons. No significant differences were observed in the pre- and post-diet leptin and ghrelin levels in either group (*p* > 0.05). Between-group comparisons of change scores (Δ) showed no significant differences for leptin (*p* = 0.229) or ghrelin (*p* = 0.532).

The comparison of pre- and post-diet PSQI and MEQ scores according to the diet groups is presented in Table 5. The MEQ total score significantly increased in the MedDiet group after the intervention (*p* < 0.05), whereas no significant change was observed in the TRE group. No significant differences were detected in PSQI total scores or MEQ chronotype distribution between the pre- and post-diet assessments in either group (*p* > 0.05).

As the assumptions of normality and variance homogeneity were met, a mixed-design ANOVA test was conducted for the PSQI total and MEQ total scores, and the results are presented in Table 6. For PSQI, the analysis revealed a significant main effect of time (pre/post), indicating that the PSQI scores decreased from pre- to post-intervention regardless of diet type (F = 11.370, *p* = 0.002, η^2^p = 0.275, observed power = 0.960). The interaction effect between time and diet type (F = 1.392, *p* = 0.247, η^2^p = 0.044) and the main effect of diet type (F = 2.756, *p* = 0.107, η^2^p = 0.084) were not statistically significant. These results indicate that the effect of diet type on changes over time was not significant and that, overall, the diets did not result in a meaningful difference in the measured outcomes. The effect sizes support the finding that the time effect was moderate, whereas the effects of diet type and the interaction were minor. The high observed power of the analysis indicates that the time effect is not only statistically significant but also clinically meaningful. For the MEQ total scores, the main effect of time was not significant (F = 1.226, *p* = 0.276, η^2^p = 0.037). This indicates that there were no significant differences in MEQ scores over time, regardless of diet type. In addition, neither the interaction effect between time and diet type (F = 1.226, *p* = 0.276, η^2^p = 0.037) nor the main effect of diet type (F = 1.064, *p* = 0.310, η^2^p = 0.032) was statistically significant. These results suggest that the type of diet did not result in meaningful differences in MEQ scores over time, and all associated effect sizes were minor. In contrast, for PSQI scores, a decrease was observed in both diet groups from the pre-diet to the post-diet period. This reduction indicates an overall improvement in sleep quality. The mixed-design ANOVA confirmed that the main effect of time was statistically significant (*p* = 0.002).

## 4. Discussion

MS prevalence has been steadily increasing worldwide. Recent literature has highlighted the potential impact of dietary interventions on sleep patterns, chronotype, and appetite-related parameters in patients with MS, emphasizing the need for further research to elucidate their long-term effectiveness [37]. The primary aim of this two-arm randomized, parallel-group comparative pilot trial was to evaluate the effects of the MedDiet and TRE on anthropometric parameters, lipid profile, sleep quality, appetite-related hormones, and chronotype in females with RRMS. Both interventions were associated with significant reductions in anthropometric measures during the intervention period, whereas trends toward favorable changes in LDL cholesterol and total cholesterol, together with an increase in self-reported MEQ scores, were observed in the MedDiet group. No significant changes were detected in PSQI scores, leptin, or ghrelin concentrations. This study is among the first to directly compare the effects of MedDiet and TRE on anthropometric, biochemical, chronotype, sleep, and appetite-related outcomes in females with RRMS.

MS is an immune-mediated neurodegenerative disease wherein inflammation, oxidative stress, and metabolic dysregulation contribute to MS pathogenesis. Dietary factors may influence both the mechanisms underlying MS pathology and the degree of activity [38]. In patients with neurodegenerative diseases, including MS, comprehensive nutritional assessment and appropriate nutritional treatment are crucial. Dietary patterns that are anti-inflammatory and support metabolic balance have been proposed as complementary strategies in MS management [39]. In this context, the reductions in BMI, WC, and HC observed in both groups in this study suggest that these dietary interventions were associated with favorable anthropometric changes in patients with RRMS.

MedDiet, broadly investigated for its impact on various chronic diseases, and TRE, increasingly recognized for its health effects, represent two distinct dietary approaches with potential implications for disease management. The MedDiet is characterized by a high intake of plant-based foods, with olive oil as the primary fat source; moderate consumption of fish, poultry, and dairy products; and a rich supply of polyphenols and flavonoids [40,41]. The MedDiet’s antioxidant, anti-inflammatory, and immunomodulatory properties have been associated with reduced risk of noncommunicable diseases and MS [42,43,44]. Increased adherence to the MedDiet has been linked to slower disease progression, lower disease severity, and improved quality of life in patients with MS [45,46,47]. Conversely, TRE, which limits daily food intake to a specific time window of <12 h, has gained attention for its potential to align with the body’s circadian rhythm and enhance metabolic regulation [48]. Previous studies have reported that TRE may reduce inflammation and oxidative stress, improve cardiometabolic health, and alleviate symptom burden [19,49]. Through mechanisms including enhanced autophagy, improved insulin sensitivity, and reduced oxidative damage, TRE has been shown to support cognitive function and mitochondrial health, both of which are frequently compromised in MS and other neurodegenerative disorders [12,13,16,17,38,50]. In the present study, both the MedDiet and TRE groups demonstrated improved dietary profiles, characterized by increased protein, fiber, and water intake and reduced fat intake, accompanied by significant reductions in BMI, WC, and HC. Both groups were prescribed diet plans designed to be eucaloric based on estimated energy requirements calculated using the Harris–Benedict equation, and the 12-week average energy intake did not differ significantly from baseline values (MedDiet: *p* = 0.108; TRE: *p* = 0.308). Similarly, carbohydrate intake (g/day) remained unchanged in both groups, whereas protein intake increased, and fat intake decreased significantly, indicating that the observed dietary changes primarily reflected a shift in macronutrient composition rather than a reduction in reported energy intake. However, the observed moderate weight loss (Cohen’s d: MedDiet = 0.659, TRE = 0.540) suggests that participants may have experienced an unintended negative energy balance during the intervention. This may reflect limitations of self-reported dietary assessment methods, which are known to be susceptible to underreporting and measurement error, spontaneous reductions in energy intake associated with increased protein and fiber consumption, or behavioral changes during the intervention period. Several factors may have contributed to the observed discrepancy. However, because objective measures of dietary adherence and energy expenditure were not available, the relative contribution of each factor to the observed weight loss could not be determined. Consequently, the trends toward favorable changes in the lipid parameters observed in the MedDiet group may reflect the combined effects of dietary composition and an unintended negative energy balance and therefore cannot be attributed exclusively to diet quality. Although no significant differences were observed between groups, both dietary interventions resulted in increased protein, fiber, and water intake together with reduced fat intake, while carbohydrate intake remained stable. A systematic review has recommended limiting saturated fatty acid intake to reduce symptom burden during the inflammatory process of MS [51]. A previous study investigating the association between MS-related symptoms and nutrition found that participants who consumed more than one serving of red meat or products per day had significantly higher symptom scores, whereas those who consumed more than three servings of fish per week had lower symptom scores [52]. In the present study, the ratio of energy derived from fat decreased in both groups after the dietary intervention, whereas dietary PUFA intake showed no change.

It has been reported that 70% of patients with RRMS are at nutritional risk; 20% are malnourished; and 50% are overnourished [53]. Previous studies have demonstrated beneficial effects of both TRE and the MedDiet on body weight and WC [54,55,56]. Consistent with these findings, both intervention groups in the present study exhibited significant reductions in BMI, WC, and HC. Although the prescribed diets were designed to be eucaloric and no significant reduction in reported energy intake was observed, moderate weight loss occurred in both groups. Previous evidence suggests that TRE may support weight management through changes in meal timing and eating patterns, whereas the MedDiet may contribute to metabolic health through its nutrient composition and dietary quality [57]. Therefore, the anthropometric changes observed in the present study may reflect the combined effects of dietary modifications, increased dietary awareness, and possible unintended negative energy balance during the intervention. Accordingly, it is not possible to distinguish the independent effects of the dietary interventions from those of a potential negative energy balance.

Initially, research on circadian rhythm focused on the structure of the biological clock and the investigation of circadian disruptions in several diseases; however, the concept of enhancing and sustaining the circadian rhythm for disease management has recently gained significance [58]. Although circadian rhythm disruption has been associated with MS, it remains unknown whether these disruptions are the cause or result of MS-related neuroinflammation [19,20]. Chronotherapeutic approaches, including TRE, have been proposed to influence circadian-related processes through the timing of food intake [19]. Moreover, fasting-mimicking diets and classical ketogenic diets have shown potential benefits in RRMS treatment [59]. Recent studies have also identified a possible association between chronotype and MedDiet [10,11,12,13]. Evening-type individuals tend to consume fewer whole grains, fish, vegetables, and fruits and consume more alcoholic beverages and desserts compared with the morning-type individuals [15]. In a different study, individuals with the morning chronotype exhibited more favorable attitudes toward nutrition and greater compliance with the MedDiet than those with the intermediate and evening chronotypes [60]. In the present study, MEQ scores increased significantly in the MedDiet group following the intervention (*p* < 0.05), whereas no significant changes were observed in the TRE group. Although this increase was not sufficient to alter chronotype classification, it suggests a shift toward a more morning-oriented chronotype. The absence of significant changes in the TRE group may be related to baseline characteristics of the participants, duration of the intervention, or differences in individual responses to the intervention. Previous studies have suggested that bioactive components of the MedDiet, including polyphenols, omega-3 fatty acids, and other micronutrients, may be associated with pathways involved in sleep–wake regulation and chronotype preference [61,62,63]. However, because chronotype was assessed using a self-reported questionnaire and objective circadian markers were not evaluated, these mechanisms could not be evaluated in the present study. Therefore, the findings should be interpreted as reflecting self-reported changes in chronotype preference, rather than direct evidence of circadian rhythm modulation. Overall, these findings suggest a potential association between adherence to the MedDiet and self-reported chronotype preference in females with RRMS, warranting further investigation using objective measures of circadian function.

Patients with MS frequently experience sleep disorders. A previous study revealed that 74% of patients with MS exhibited sleep-related issues, including insomnia, sleep-disordered breathing, and circadian rhythm disturbance [64]. Another study conducted on 51 patients with RRMS reported that poor sleep quality was significantly associated with reduced cognitive performance, lower functional capacity, and poorer health-related quality of life, including impairments in physical, psychological, and occupational functioning [65]. Previous studies have suggested that both dietary composition and meal timing may influence sleep-related outcomes. Adherence to the MedDiet has been associated with better sleep quality, potentially owing to its high content of fruits, vegetables, and other bioactive compounds [66,67]. Likewise, chrono-nutritional approaches such as TRE have been investigated for their potential effects on metabolic health and sleep-related parameters [68,69,70]. In the present study, all participants had good sleep quality at baseline, with PSQI scores below the clinical threshold of 5. Although PSQI scores decreased slightly in both groups following the intervention, no significant differences were observed between the dietary interventions. The significant effect of time suggests a modest improvement in self-reported sleep quality throughout the intervention period regardless of dietary approach. However, in the absence of a non-intervention control group, this improvement cannot be attributed specifically to either dietary intervention, and may partly reflect non-specific effects associated with study participation. Furthermore, the favorable baseline sleep quality of the study population may have limited the potential for clinically meaningful improvement. In addition, a longer intervention period may be required to determine whether MedDiet and TRE exert differential effects on sleep-related outcomes. Furthermore, sleep quality and chronotype were assessed using self-reported questionnaires (PSQI and MEQ), without objective measures of circadian function. Therefore, the findings should be interpreted as reflecting self-reported changes in sleep quality and chronotype preference rather than objective alterations in circadian biology.

Cardiovascular risk factors are prevalent among patients with MS [68]. Increased oxidative stress has been associated with endothelial dysfunction, arterial remodeling and stiffness, and atherosclerosis [71,72,73]. A previous study revealed that patients with MS had significantly higher serum total cholesterol and TG levels and significantly lower serum HDL cholesterol levels than controls. Moreover, obesity in RRMS has been associated with clinical disability, increased central inflammation, elevated cerebrospinal fluid levels of interleukin (IL)-6 and leptin, decreased IL-13, and an unfavorable lipid profile, including higher serum TG level and total cholesterol/HDL ratio. These metabolic alterations have been linked to greater disease severity at diagnosis [74]. Evidence from previous studies suggests that the MedDiet may help mitigate these risks. A randomized controlled trial reported that the levels of inflammatory markers dramatically decreased following the MedDiet [75]. In the present study, serum LDL cholesterol and total cholesterol levels showed favorable changes in the MedDiet group before correction for multiple comparisons; however, these findings did not remain statistically significant after correction. The magnitude of these changes was modest, and all lipid parameters remained within reference ranges. Similarly, another randomized controlled trial investigating the mid- and long-term effects of the MedDiet revealed improvements in leptin, C-reactive protein, blood pressure, and pro-inflammatory markers at 6- and 12-month assessments [46]. Taken together, our findings suggest a trend toward favorable changes in lipid parameters following the MedDiet; however, these findings should be interpreted cautiously because they did not remain statistically significant after correction for multiple comparisons.

Adipocytes produce leptin in amounts proportional to their volume, and leptin plays a crucial role in regulating appetite and maintaining energy balance [76]. Several intervention studies have reported that hypocaloric diets based on the MedDiet significantly decreased the plasma concentrations of inflammatory biomarkers, including leptin [77,78,79]. Ghrelin, another appetite-related hormone, increases during fasting and decreases immediately after a meal [80]. Low fasting ghrelin levels are associated with insulin resistance and obesity [81,82,83,84,85]. A previous study showed that ghrelin levels in-creased following an 18-month MedDiet [86], whereas a meta-analysis reported that individuals adhering to a fasting diet exhibited decreased leptin and ghrelin levels compared with the control group [87]. Contrary to these findings, no significant within-group changes were observed in leptin or ghrelin levels following either dietary intervention. Furthermore, comparison of change scores (Δ = post-intervention − baseline) between the MedDiet and TRE groups revealed no significant differences in the magnitude of hormonal changes, suggesting that neither dietary approach exerted a greater effect on these appetite-related hormones over the 12-week intervention period. These findings may be explained by the relatively short intervention duration, individual variability in hormone concentrations, prolonged fasting periods, or flexibility in meal timing. Furthermore, experimental and observational studies have reported that acute- and long-term sleep restriction can decrease leptin and increased ghrelin levels [88,89]. Leptin and ghrelin have been identified as potential neuroendocrine mediators in the pathway linking inadequate sleep to obesity, due to the increased appetite associated with altered levels of these hormones [89]. Given that our participants already had good baseline sleep quality and experienced only minor changes in sleep-related parameters, this may have contributed to the absence of detectable changes in leptin and ghrelin levels. Leptin and ghrelin do not have universally accepted standard reference ranges because their circulating concentrations are highly influenced by multiple biological and methodological factors, including substantial diurnal variation, strong dependence on sex and adiposity, and significant variability across measurement techniques (RIA, ELISA, LC-MS). In addition, manufacturers of commercial assay kits provide different reference intervals, further preventing the establishment of a single standardized range. Therefore, the evaluation focused primarily on the comparison of intervention-induced changes between groups rather than on absolute hormone concentrations or non-standardized reference ranges. Together with the absence of significant within-group changes, these findings suggest that the 12-week MedDiet and TRE interventions in the present study did not result in measurable short-term alterations in circulating leptin or ghrelin concentrations in women with RRMS.

This study has several limitations that should be considered. First, the relatively small sample size, inclusion of only female participants, and single-center design may limit the generalizability of the findings. Although the study met the sample size requirement identified by the a priori power analysis, larger studies may help further clarify the magnitude and consistency of the observed effects. Despite the application of the Benjamini–Hochberg correction for multiple comparisons, the relatively small sample size of this pilot study may increase the possibility of both Type I and Type II statistical errors. Accordingly, the findings should be interpreted with appropriate caution until confirmed in larger, adequately powered studies. Second, the absence of a non-intervention control group makes it difficult to determine whether the observed changes were attributable solely to the dietary interventions or were partially influenced by non-dietary factors, such as increased dietary awareness, behavioral modifications, or participation in a clinical study. This limitation should be considered when interpreting the observed effects of the dietary interventions. The intervention period was limited to 12 weeks. Although dietary interventions of similar duration have been commonly used in studies involving individuals with multiple sclerosis [59,90,91,92,93], longer interventions have been reported to provide additional information regarding sustained metabolic and biochemical adaptations [94,95]. Therefore, longer follow-up periods may be required to fully evaluate long-term changes in lipid metabolism, glycemic markers, appetite-related hormones, and other clinical outcomes. Another limitation relates to the assessment of dietary intake and adherence. Although individualized diet plans were designed to be eucaloric based on estimated energy requirements and no statistically significant changes in reported energy intake were observed, both intervention groups experienced significant reductions in anthropometric measures. This discrepancy may reflect several factors, including limitations inherent to self-reported dietary assessment methods, spontaneous reductions in energy intake associated with changes in dietary composition, behavioral modifications during study participation, or unintended negative energy balance. Because objective measures of dietary adherence and energy expenditure were not available, the relative contribution of these factors could not be determined. Consequently, the independent contributions of dietary composition and potential negative energy balance to the observed anthropometric and metabolic changes could not be distinguished. A further limitation is that sleep quality and chronotype were assessed exclusively using self-reported questionnaires (PSQI and MEQ), without the inclusion of objective circadian biomarkers such as melatonin, cortisol, or other physiological indicators of circadian function. Consequently, the findings should be interpreted as self-reported changes in sleep quality and chronotype preference rather than objective alterations in circadian biology. Finally, only one form of intermittent fasting (TRE) was evaluated, preventing comparisons with other intermittent fasting protocols. Given the exploratory nature of the present pilot study, future studies should include larger and more diverse populations, longer intervention periods, objective measures of dietary adherence and circadian function, and additional biological outcomes such as inflammatory markers, oxidative stress biomarkers, microbiota composition, and neuroimaging parameters to better clarify the mechanisms underlying dietary effects in multiple sclerosis.

## 5. Conclusions

This randomized comparative pilot study evaluated the effects of MedDiet and TRE on anthropometric, biochemical, sleep-related, chronotype, and appetite-related outcomes in females with RRMS. Both interventions were associated with changes in anthropometric measures, whereas modest favorable changes in lipid parameters and higher MEQ scores were observed in the MedDiet group. No significant changes were detected in self-reported sleep quality, leptin, or ghrelin concentrations. The observed changes in chronotype should be interpreted as self-reported changes in chronotype preference rather than direct evidence of circadian rhythm modulation. Given the pilot nature of the study, the absence of a non-intervention control group, and the relatively small sample size, further trials with longer follow-up periods and objective circadian and metabolic measures are needed to clarify the long-term effects of these dietary interventions. Overall, the present findings provide preliminary evidence supporting the potential role of nutritional interventions as complementary approaches in the management of RRMS and may help generate hypotheses for future clinical studies.

## Figures and Tables

**Table 1:** Demographic and clinical characteristics of the participants according to the diet groups.

	Diet Groups				Total		Effect Size	Test Statistic	*p*	95% CI	
	MedDiet (*n* = 17)		TRE (*n* = 17)								
	*n*	%	*n*	%	*n*	%				Lower	Upper
Age (year) (mean ± SD)	34.06 ± 8.55		34.65 ± 8.23		34.35 ± 8.27		d = 0.070	−0.204	0.839 ^t^	−6.45	5.28
Age at Diagnosis (year) (mean ± SD)	27 (17–43)		22 (18–38)		25 (17–43)		d = 0.044	−1.225	0.220 ^m^	−2.00	6.00
Marital Status											
Married	12	70.6	12	70.6	24	70.6	V = 0.000	-	1.000 ^f^	−0.37	0.37
Single	5	29.4	5	29.4	10	29.4					
Education											
Middle and High School Bachelor and Postgraduate	7 10	41.2 58.8	9 8	52.9 47.1	16 18	47.1 52.9	V = 0.118	0.118	0.731 ^f^	−0.45	0.22
Occupation											
Housewife	6	35.3	11	64.7	17	50	V = 0.458	6.582	0.075 ^f^		
Student	3	17.6	0	0	3	8.8				−0.46	0.22
Health Professional	6	35.3	2	11.8	8	23.5					
Teacher	2	11.8	4	23.5	6	17.6					
Physical Activity											
Yes	11	64.7	11	64.7	22	64.7	V = 0.000	-	1.000 ^f^	−0.35	0.35
No	6	35.3	6	35.3	12	35.3					
Smoking											
Yes	5	29.4	1	5.9	6	17.6	V = 0.309	-	0.175 ^f^	0.05	0.75
No	12	70.6	16	94.1	28	82.4					
Alcohol											
Yes	1	5.9	1	5.9	2	5.9	V = 0.000	-	1.000 ^f^	−0.71	0.71
No	16	94.1	16	94.1	32	94.1					

MedDiet, Mediterranean diet; TRE, time-restricted eating; *n*, number; SD, standard deviation; d, Cohen’s d; t, independent two-sample *t*-test; m, Mann–Whitney U test; f, Fisher’s exact test; V, Cramer’s V; CI, Confidence Interval.

**Table 2:** Pre- and post-diet anthropometric measurements of the participants according to the diet groups.

		Diet Groups			Cohen’s d	Test Statistic	*p*	95% CI	
		MedDiet	TRE	Total					
		(*n* = 17)	(*n* = 17)						
		Mean ± SD	Mean ± SD	Mean ± SD				Lower	Upper
Height (cm)		163.47 ± 6.77	163.47 ± 5.08	163.47 ± 5.89	0.000	0.000	1.000 ^t^	−4.18	4.18
Pre-diet BMI (kg/m^2^)		23.96 ± 2.02	23.81 ± 1.81	23.88 ± 1.89	0.081	0.236	0.815 ^t^	−1.18	1.49
Post-diet BMI (kg/m^2^)		23.57 ± 1.66	23.4 ± 1.35	23.49 ± 1.49	0.112	0.325	0.748 ^t^	−0.88	1.22
Test Statistic		2.591	2.263						
*p* ^e^ (BH threshold) ^a^		0.020 (0.030)	0.038 (0.030)						
Cohen’s d		0.628	0.549						
95% CI	Lower	0.071	0.026						
	Upper	0.711	0.783						
Pre-diet WC (cm)		82.35 ± 7.98	80.59 ± 7.83	81.47 ± 7.84	0.224	0.650	0.520 ^t^	−3.76	7.29
Post-diet WC (cm)		81 ± 7.05	79.59 ± 6.96	80.29 ± 6.94	0.202	0.587	0.561 ^t^	−3.48	6.31
Test Statistic		3.159	2.608						
*p* ^e^ (BH threshold) ^a^		0.006 (0.010)	0.019 (0.020)						
Cohen’s d		0.766	0.633						
95% CI	Lower	0.445	0.187						
	Upper	2.261	1.813						
Pre-diet HC (cm)		101 ± 8.61	103 ± 6.84	102 ± 7.72	0.257	−0.750	0.459 ^t^	−7.43	3.43
Post-diet HC (cm)		99.82 ± 7.65	101.47 ± 5.62	100.65 ± 6.66	0.247	−0.715	0.480 ^t^	−6.34	3.04
Test Statistic		2.582	2.889						
*p* ^e^ (BH threshold) ^a^		0.020 (0.040)	0.011 (0.010)						
Cohen’s d		0.626	0.701						
95% CI	Lower	0.211	0.407						
	Upper	2.142	2.652						
Pre-diet WHR		0.81 ± 0.07	0.78 ± 0.05	0.8 ± 0.07	0.589	1.648	0.109 ^t^	−0.01	0.08
Post-diet WHR		0.81 ± 0.07	0.78 ± 0.06	0.79 ± 0.06	0.479	1.358	0.184 ^t^	−0.01	0.07
Test Statistic		1.289	−0.418						
*p* ^e^		0.216	0.681						
Cohen’s d		0.313	0.101						
95% CI	Lower	−0.003	−0.015						
	Upper	0.012	0.010						
Pre-diet weight (kg)		64.19 ± 8.03	63.67 ± 6.27	63.93 ± 7.10	0.073	0.212	0.834 ^t^	−4.51	5.56
Post-diet weight (kg)		63.09 ± 7.01	62.58 ± 4.99	62.84 ± 5.99	0.084	0.245	0.808 ^t^	−3.74	4.76
Test Statistic		2.716	2.226						
*p* ^e^ (BH threshold) ^a^		0.015 (0.020)	0.041 (0.040)						
Cohen’s d		0.659	0.540						
95% CI	Lower	0.242	0.052						
	Upper	1.964	2.130						

MedDiet, Mediterranean diet; TRE, time-restricted eating; WC, waist circumference; HC, hip circumference; WHR, waist/hip ratio; BMI, body mass index; *n*, number; SD, standard deviation; t, independent two-sample *t*-test; e, paired two-sample *t*-test; d, Cohen’s d; a, Benjamini–Hochberg (BH) threshold; CI, Confidence Interval.

**Table 3:** Pre-diet and 12-week average diet mean energy and macronutrient intake of participants according to diet groups.

		Diet Groups			Effect Size	Test Statistic	*p*	95% CI	
		MedDiet	TRE	Total					
		(*n* = 17)	(*n* = 17)						
		Mean ± SD	Mean ± SD	Mean ± SD				Lower	Upper
Pre-diet energy (kcal/day) (mean ± SD)		1849.53 ± 108.75	1830.94 ± 124.06	1840.23 ± 115.26	0.159 ^d^	0.465	0.645 ^t^	−62.91	100.10
12-week average energy (kcal/day) (mean ± SD)		1797.36 ± 126.93	1798.3 ± 77.7	1797.83 ± 103.63	0.009 ^d^	−0.026	0.979 ^t^	−74.46	72.59
Test Statistic		1.701	1.053						
*p* ^e^		0.108	0.308						
Cohen’s d		0.413	0.255						
95% CI	Lower	−12.860	−33.080						
	Upper	117.190	98.350						
Pre-diet water (mL/day) (mean ± SD)		1477.69 ± 194.14	1458.18 ± 148.39	1467.94 ± 170.44	0.113 ^d^	0.329	0.744 ^t^	−101.22	140.23
12-week average water (mL/day) (mean ± SD)		1744.08 ± 298.46	1760.33 ± 292.35	1752.2 ± 291.03	0.055 ^d^	−0.160	0.874 ^t^	−22.65	190.15
Test Statistic		−3.324	−3.234						
*p* ^e^ (BH threshold) ^a^		0.004 (0.029)	0.005 (0.029)						
Cohen’s d		0.806	0.784						
95% CI	Lower	−436.300	−500.240						
	Upper	−96.480	−104.060						
Pre-diet protein (g/day) (mean ± SD)		73.26 ± 13.67	72.22 ± 16.51	72.74 ± 14.94	0.069 ^d^	0.200	0.843 ^t^	−9.55	11.63
12-week average Protein (g/day) (mean ± SD)		98.46 ± 14.84	96.07 ± 10.36	97.26 ± 12.66	0.187 ^d^	0.544	0.590 ^t^	−6.55	11.33
Test Statistic		5.788	5.488						
p (BH) ^a^		0.001 ^e^ (0.017)	0.001 ^e^ (0.017)						
Effect size		1.404 ^d^	1.331 ^d^						
95% CI	Lower	15.97	14.64						
	Upper	34.43	33.07						
Pre-diet protein (%) (mean ± SD)		16.35 ± 2.76	16.12 ± 3.55	16.24 ± 3.13	0.072 ^d^	0.216	0.831 ^t^	−1.99	2.46
12-week average protein (%) (mean ± SD)		22.53 ± 2.83	21.94 ± 2.46	22.24 ± 2.63	0.223 ^d^	0.647	0.523 ^t^	−1.27	2.44
Test Statistic		−6.264	−5.541						
*p* ^e^ (BH threshold) ^a^		0.001 (0.007)	0.001 (0.007)						
Cohen’s d		1.519	1.344						
95% CI	Lower	−8.270	−8.050						
	Upper	−4.090	−3.600						
Pre-diet fat (g/day) (mean ± SD)		83.45 ± 14.57	80.48 ± 11.84	81.96 ± 13.16	0.224 ^d^	0.652	0.519 ^t^	−6.31	12.25
12-week average fat (g/day) (mean ± SD)		57.33 ± 8.61	56.35 ± 5.41	56.83 ± 7.10	0.138 ^d^	0.403	0.689 ^t^	−4.03	6.02
Test Statistic		−5.956	−9.36						
*p* (BH) ^a^		0.001 ^e^ (0.033)	0.001 ^e^ (0.033)						
Effect size		−1.445 ^d^	−2.27 ^d^						
95% CI	Lower	−35.41	−29.61						
	Upper	−16.82	−18.67						
Pre-diet fat (%) (mean ± SD)		40.29 ± 6.91	39.06 ± 4.81	39.68 ± 5.9	0.207 ^d^	0.605	0.550 ^t^	−2.93	5.40
12-week average fat (%) (mean ± SD)		28.53 ± 3.86	28.18 ± 2.32	28.35 ± 3.14	0.110 ^d^	0.323	0.749 ^t^	−1.87	2.58
Test Statistic		5.434	9.680						
*p* ^e^ (BH threshold) ^a^		0.001 (0.014)	0.001 (0.014)						
Cohen’s d		1.318	2.348						
95% CI	Lower	7.180	8.500						
	Upper	16.360	13.270						
Pre-diet CHO (g/day) (mean ± SD)		196.43 ± 38.76	199.65 ± 30.22	198.04 ± 34.26	−0.093 ^d^	−0.270	0.789 ^t^	−27.51	21.06
12-week average CHO (g/day) (mean ± SD)		212.22 ± 21.35	215.28 ± 17.56	213.75 ± 19.31	−0.157 ^d^	−0.457	0.651 ^t^	−16.72	10.60
Test Statistic		1.262	1.790						
*p*		0.225 ^e^	0.092 ^e^						
Effect size		0.306 ^d^	0.434 ^d^						
95% CI	Lower	−10.74	−2.88						
	Upper	42.32	34.13						
Pre-diet CHO (%) (mean ± SD)		43.42 ± 7.82	44.71 ± 6.22	44.06 ± 6.99	0.269 ^d^	−0.778	0.436 ^m^	−6.23	3.64
12-week average CHO (%) (mean ± SD)		48.77 ± 3.90	49.59 ± 3.02	49.18 ± 3.46	0.054 ^d^	−0.158	0.874 ^m^	−3.26	1.61
Test Statistic		−2.086	−2.910						
*p* ^e^ (BH threshold) ^a^		0.053	0.010 (0.036)						
Cohen’s d		−0.506	−0.706						
95% CI	Lower	−10.790	−8.440						
	Upper	0.090	−1.330						
Pre-diet PUFA (g/day) (mean ± SD)		14.91 ± 4.2	12.58 ± 3.38	13.74 ± 3.94	0.611 ^d^	1.784	0.084 ^t^	−0.33	5.00
12-week average PUFA (g/day) (mean ± SD)		13.55 ± 2.8	14.21 ± 2.35	13.88 ± 2.57	0.255 ^d^	−0.747	0.460 ^t^	−2.47	1.14
Test Statistic		1.038	−1.420						
*p* ^e^		0.315	0.175						
Cohen’s d		0.252	0.344						
95% CI	Lower	−1.420	−4.070						
	Upper	4.150	0.810						
Pre-diet fiber (g/day) (median [min–max])		18.9 (11.9–57.6)	19.7 (14.3–27.6)	19.35 (11.9–57.6)	0.038 ^rb^	139.00	0.863 ^m^	−3.80	4.60
12-week average fiber (g/day) (median [min–max])		38.9 (28.3–56.4)	39.2 (28.59–47.7)	39.05 (28.3–56.4)	0.024 ^rb^	141.00	0.918 ^m^	−4.20	6.8
Test Statistic		−3.101	−3.622						
*p* ^w^ (BH threshold) ^a^		0.002 (0.021)	0.001 (0.021)						
Cohen’s d		2.282	3.677						
95% CI	Lower	−25.900	−23.390						
	Upper	−10.700	−15.790						

MedDiet, Mediterranean diet; TRE, time-restricted eating; CHO, carbohydrate; PUFA, polyunsaturated fatty acid; g, gram; kcal, kilocalorie; *n*, number; SD, standard deviation; t, independent two-sample *t*-test; m, Mann–Whitney U test; e, paired two-sample *t*-test; w, Wilcoxon test; d, Cohen’s d; a, Benjamini–Hochberg (BH) threshold; CI, Confidence Interval; rb, rank biserial correlation.

**Table 4:** Pre- and post-diet biochemical markers of the participants according to the diet groups.

		Diet Groups			Effect Size	Test Statistic	*p*	95% CI	
		MedDiet	TRE	Total					
		(*n* = 17)	(*n* = 17)						
		Mean ± SD	Mean ± SD	Mean ± SD				Lower	Upper
Pre-diet FSG (mg/dL) (mean ± SD)		86.76 ± 7.55	85.18 ± 6.6	85.97 ± 7.03	0.223	0.653	0.518 ^t^	−3.37	6.54
Post-diet FSG (mg/dL) (mean ± SD)		86.82 ± 5.03	85.35 ± 6.64	86.09 ± 5.85	0.25	0.728	0.472 ^t^	−2.65	5.59
Test Statistic		−0.040	−0.090						
*p* ^e^		0.969	0.929						
Cohen’s d		0.010	0.022						
95% CI	Lower	−3.19	−4.33						
	Upper	3.08	3.98						
Pre-diet TG (mg/dL) (mean ± SD)		95.47 ± 39.67	97 ± 26.11	96.24 ± 33.08	0.046	−0.133	0.895 ^t^	−25.14	22.08
Post-diet TG (mg/dL) (mean ± SD)		99.47 ± 35.25	92.47 ± 25.99	95.97 ± 30.7	0.226	0.659	0.515 ^t^	−14.64	28.69
Test Statistic		−0.594	1.259						
*p* ^e^		0.561	0.226						
Cohen’s d		0.144	0.305						
95% CI	Lower	−10.50	−1.50						
	Upper	8.50	7.00						
Pre-diet HDL (mg/dL) (median [min–max])		60 (40–82)	54 (45–82)	58 (40–82)	0.380	−1.088	0.276 ^m^	−4.08	13.96
Post-diet HDL (mg/dL) (median [min–max])		56 (45–79)	56 (45–82)	56 (45–82)	0.361	−1.036	0.300 ^m^	−4.60	11.78
Test Statistic		−0.207	−0.900						
*p* ^w^		0.836	0.368						
Cohen’s d		0.101	0.447						
95% CI	Lower	−2.16	−2.50						
	Upper	4.16	2.00						
Pre-diet LDL (mg/dL) (mean ± SD)		101.88 ± 21.07	95.24 ± 30.22	98.56 ± 25.87	0.255	0.744	0.462 ^t^	−11.55	24.85
Post-diet LDL (mg/dL) (mean ± SD)		99.12 ± 20.87	93.12 ± 25.62	96.12 ± 23.21	0.257	0.749	0.460 ^t^	−10.32	22.32
Test Statistic		2.233	0.576						
*p* ^e^ (BH threshold) ^a^		0.040 (0.005)	0.573						
Cohen’s d		0.542	0.140						
95% CI	Lower	0.14	−0.50						
	Upper	5.39	8.00						
Pre-diet total cholesterol (mg/dL) (median [min–max])		178 (134–200)	167 (118–248)	172.5 (118–248)	0.398	1.162	0.256 ^m^	−8.88	31.82
Post-diet total cholesterol (mg/dL) (median [min–max])		173 (120–198)	164 (121–258)	168.5 (120–258)	0.506	−1.43	0.153 ^m^	−11.47	30.18
Test Statistic		2.122	0.95						
*p*^w^ (BH threshold) ^a^		0.050 (0.011)	0.169						
Cohen’s d		0.515	0.397						
95% CI	Lower	0.01	−2.00						
	Upper	10.47	17.00						
ΔLeptin (mean ± SD) (ng/mL)		−0.02 ± 0.24	−0.13 ± 0.28	−0.07 ± 0.27	0.421 ^d^	1.226	0.229 ^t^	−0.07	0.30
Pre-diet leptin (mean ± SD) (ng/mL)		7.63 ± 0.63	8.02 ± 0.75	7.82 ± 0.71	−0.562	−1.639	0.111 ^t^	−0.87	0.09
Post-diet leptin (mean ± SD) (ng/mL)		7.61 ± 0.58	7.89 ± 0.57						
Test Statistic		−0.288	−1.856						
*p* ^e^		0.777	0.082						
Effect size		−0.07 ^d^	−0.45 ^d^						
95% CI	Lower	−0.142	−0.275						
	Upper	0.108	0.018						
ΔGhrelin (mean ± SD) (ng/mL)		−1.06 ± 22.39	−5.48 ± 18.10	−3.28 ± 20.18	0.217 ^d^	0.632	0.532 ^t^	−9.81	18.64
Pre-diet ghrelin (mean ± SD) (ng/mL)		730.64 ± 39.54	748.22 ± 49.63	739.43 ± 45.07	−0.392	−1.143	0.262 ^t^	−48.93	13.76
Post-diet ghrelin (mean ± SD) (ng/mL)		729.57 ± 35.21	742.74 ± 40.22						
Test Statistic		−0.196	−1.248						
*p* ^e^		0.847	0.230						
Effect size		−0.047 ^d^	−0.303 ^d^						
95% CI	Lower	−12.58	−14.784						
	Upper	10.45	3.829						

MedDiet, Mediterranean diet; TRE, time-restricted eating; FSG, fasting serum glucose; HDL, high-density lipoprotein; LDL, low-density lipoprotein; TG, triglyceride; *n*, number; SD, standard deviation; t, independent two-sample *t*-test; m, Mann–Whitney U test; e, paired two-sample *t*-test; w, Wilcoxon test; d, Cohen’s d; ng, nanogram; mL, milliliter; a, Benjamini–Hochberg (BH) threshold; CI, Confidence Interval.

**Table 5:** Pre- and post-diet PSQI and MEQ scores of the participants according to the diet groups.

		Diet Groups		Effect Size	Test Statistic	*p*	95% CI	
		MedDiet	TRE					
		(*n* = 17)	(*n* = 17)				Lower	Upper
Pre-Diet MEQ Total Score (mean ± SD)		52.41 ± 12.33	56.71 ± 9.44	d = 0.392	−1.140	0.263 ^t^	−12.08	3.14
Post-Diet MEQ Total Score (mean ± SD)		53.76 ± 12.76	57.71 ± 9.5	d = 0.351	−1.021	0.315 ^t^	−0.55	1.84
Test Statistic		−2.303	−1.738					
*p* ^e^		0.035	0.101					
Cohen’s d		0.559	0.422					
95% CI	Lower	−2.52	−1.50					
	Upper	−0.06	0.50					
Pre-Diet PSQI Total Score (mean ± SD)		4.35 ± 3.55	2.82 ± 1.55	d = −0.187	117.5	0.356	−1.00	−4.00
Post-Diet PSQI Total Score (mean ± SD)		3.35 ± 2.60	2.53 ± 1.62	d = 0.380	1.109	0.277	−0.70	2.35
Test Statistic		−2.10	−1.43					
*p* ^e^		0.070	0.172					
Cohen’s d		0.655	0.571					
95% CI	Lower	−2.01	−0.73					
	Upper	0.01	0.14					
		n (%)	n (%)					
Pre-Diet MEQ Chronotype Distribution								
Definitely Morning Type		1 (5.9)	2 (11.8)	V = 0.464	7.024	0.054 ^f^		
Moderately Morning Type		6 (35.3)	3 (17.6)					
Neither Type		6 (35.3)	12 (70.6)					
Moderately Evening Type		4 (23.5)	0 (0)					
Post-Diet MEQ Chronotype Distribution								
Definitely Morning Type		2 (11.8)	2 (11.8)	V = 0.383	4.846	0.211 ^f^		
Moderately Morning Type		5 (29.4)	5 (29.4)					
Neither Type		6 (35.3)	10 (58.8)					
Moderately Evening Type		4 (23.5)	0 (0)					
Test Statistic		1	1.414					
*p* ^sm^		0.317	0.157					
η^2^		0.059	0.083					

MedDiet, Mediterranean diet; TRE, time-restricted eating; PSQI, Pittsburgh Sleep Quality Index; MEQ, Morning and Evening Questionnaire; t, independent two-sample *t* test; e, paired two-sample *t* test; f, Fisher’s Exact Test; sm, Stuart–Maxwell Marginal Homogeneity test; V, Cramer’s V; d, Cohen’s d; η^2^, Eta square; *n*, number; SD, standard deviation; CI, Confidence Interval.

**Table 6:** Mixed-design ANOVA analysis of PSQI and MEQ total score values.

	Effect Size _η_^2^_p_	Statistic Test	*p _f_*
PSQI Total Score			
Time Effect	0.275	11.370	0.002
Time x Diet Type Effect	0.044	1.392	0.247
Diet Type Effect	0.084	2.756	0.107
MEQ Total Score			
Time Effect	0.037	1.226	0.276
Time x Diet Type Effect	0.037	1.226	0.276
Diet Type Effect	0.032	1.064	0.310

PSQI, Pittsburgh Sleep Quality Index; MEQ, Morning and Evening Questionnaire; η^2^p, Partial Eta square; *f*, mixed design ANOVA.

## Data Availability

The data presented in this study are available from the corresponding author upon request. The data are not publicly available due to privacy and ethical restrictions.

## Abbreviations

The following abbreviations are used in this manuscript:

ADF	Alternate-day fasting
ATADEK	Acıbadem University and Acıbadem Healthcare Institutions Medical Research Ethics Committee
BIA	Bioelectrical impedance analysis
BMI	Body mass index
CNS	Central nervous system
EDSS	Expanded Disability Status Scale
FSG	Fasting serum glucose
HC	Hip circumference
HDL	High-density lipoprotein
IF	Intermittent fasting
LDL	Low-density lipoprotein
LOD	Limit of detection
MedDiet	Mediterranean Diet
MEQ	Morningness and Eveningness Questionnaire
MS	Multiple sclerosis
PF	Periodic fasting
PSQI	Pittsburgh Sleep Quality Index
RRMS	Relapsing–remitting multiple sclerosis
TG	Triglyceride
TRE	Time-restricted eating
WC	Waist circumference
